# Modeling cancer drug response through drug-specific informative genes

**DOI:** 10.1038/s41598-019-50720-0

**Published:** 2019-10-23

**Authors:** Luca Parca, Gerardo Pepe, Marco Pietrosanto, Giulio Galvan, Leonardo Galli, Antonio Palmeri, Marco Sciandrone, Fabrizio Ferrè, Gabriele Ausiello, Manuela Helmer-Citterich

**Affiliations:** 1 Department of Biology, University of Rome “Tor Vergata”, Rome, Italy; 2 Department of Information Engineering, University of Florence, Florence, Italy; 3 Department of Pharmacy and Biotechnology, University of Bologna Alma Mater, Bologna, Italy; 4 Present Address: Celgene Institute for Translational Research Europe, Sevilla, Spain

**Keywords:** Cancer genomics, Machine learning

## Abstract

Recent advances in pharmacogenomics have generated a wealth of data of different types whose analysis have helped in the identification of signatures of different cellular sensitivity/resistance responses to hundreds of chemical compounds. Among the different data types, gene expression has proven to be the more successful for the inference of drug response in cancer cell lines. Although effective, the whole transcriptome can introduce noise in the predictive models, since specific mechanisms are required for different drugs and these realistically involve only part of the proteins encoded in the genome. We analyzed the pharmacogenomics data of 961 cell lines tested with 265 anti-cancer drugs and developed different machine learning approaches for dissecting the genome systematically and predict drug responses using both drug-unspecific and drug-specific genes. These methodologies reach better response predictions for the vast majority of the screened drugs using tens to few hundreds genes specific to each drug instead of the whole genome, thus allowing a better understanding and interpretation of drug-specific response mechanisms which are not necessarily restricted to the drug known targets.

## Introduction

The identification of genomic and molecular features that are responsible for a particular clinical outcome is one of the goals of cancer research for precision medicine^1^. Not only specific features can be discovered as biomarkers for resistance or sensitivity to a particular drug, but combinations of those same genomic and molecular features can be used to predict the effect of a drug on a patient^2^.

Recently, different studies have screened a large number of cancer cell lines with hundreds of different compounds and characterized their mutation profile, DNA methylation status, copy number alterations and gene expression in order to discover genomic features associated to a specific drug response^3–6^.

This wealth of data gave rise to a number of different methods capable of predicting the effect of a drug on different cancer cell lines, to a certain extent, using different predictive models, e.g. kernel methods, support vector regression, neural networks and random forests^7–17^. These works tackle the dual problem of improving the prediction of drug response in different cell lines while at the same time trying to identify the genomic and molecular markers underpinning specific tumors, representing a valuable resource in translational applications.

Integration of different data types has been observed to improve the prediction of drug response^4^, although possibly introducing redundancy in the information used for the predictive model^9^. From these works gene expression has emerged as the data type with the best predictive capability for the inference of drug response in different cell lines. The high dimensionality of gene expression datasets has to be taken into account when building predictive models^18^. Currently, the determination of the best combination of informative genes is a promising approach for the improvement of drug response prediction^19–21^. However this type of studies cannot be applied extensively on data generated from primary tumor samples, therefore most methods are trained and tested on small datasets of cancer cell lines, or built for few drugs, or focus on limited groups of genes which are often not effective in the prediction of drug response^22^.

In this work we show how known drug targets, and their interactome and functional context, do not generally hold a good predictive power. We therefore aimed at improving the prediction of drug response in cancer cell lines by systematically searching for informative genes to be used as features in drug-unspecific and drug-specific predictive models. Following the hypothesis that the proteome is not involved in its entirety in the response to a given drug, we selected a smaller set of informative genes that better recapitulate drug response mechanisms. We exploited two different approaches for the gene selection: first, we selected genes whose expression profiles show high variance in a dataset of nearly 1000 different cancer cell lines each treated with more than 250 different drugs, then we tried to select informative genes *ad hoc* for each screened drug, hence reducing, on average, the number of genes by two orders of magnitude. Both approaches outperform already available methods in the prediction of the resistance/sensitivity of cancer cell lines and both reduce the search space in terms of genes considered in the predictive model. We then show how the proposed approach can provide additional details on the response mechanisms of each single drug by providing both specific informative genes and unique drug-genes associations that represent a valuable starting point for follow-up experiments testing novel drug targets for anti-cancer treatment.

## Results

Gene expression has been demonstrated to hold the best predictive power for pan-cancer drug response compared to other types of data (e.g. mutations, copy number alterations and methylation data) which in turn were able to improve the predictive models in a cancer-specific fashion^4^ but could also represent a source of redundant information with gene expression^9^. In this work we show that gene expression data can be analyzed to systematically select subsets of informative genes that can be used for the prediction of pan-cancer drug response in terms of IC50 (drug concentration that reduces cell viability by 50%). We explore the predictive power of known drug targets, their close physical interactors and the pathways in which they are involved. We present two approaches which improve existing methods on a dataset of pharmacogenomics data of 961 cancer cell lines screened with 265 drugs^4^. We finally show how even though drugs of the same classes share similar profiles of resistance/sensitivity response across different cell lines, they rarely determine a drug-class-associated model able to predict the response of each drug in the same class.

### Contribution of known drug targets and of their known interaction partners to the cellular drug response

**Figure 1:**
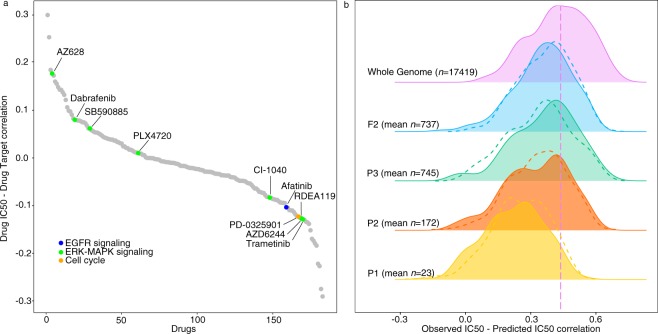
Association of drug response with pathogenic variants in the known targets and their physical and functional context. (**a**) Correlation between IC50 of 178 drugs (Y axis) and the gene expression of their known targets (the correlations for multiple targets of the same drug have been averaged). Drugs for which at least one gene mutation can discriminate resistant and sensible cell lines (Mann-Whitney U test, adjusted *p* < 0.05) have been highlighted with different colors: drugs targeting cell cycle proteins (orange), drugs targeting the EGFR signaling pathway (blue) and drugs targeting ERK-MAPK signaling pathways (green). (**b**) Performance of the ENR-based predictive model, measured as the Pearson correlation between the predicted and observed IC50, of different selections of genes (the mean number *n* of genes across the permutations is reported inset) in predicting the IC50 of 178 drugs in the whole cell line dataset; no gene set could be selected for the remaining drugs due to uncertain drug targets. The initial dataset has been permuted before the 10-fold cross validation reaching a total of 10000 training and test sets, whose results are averaged out for each drug and reported in the colored distributions. The performance of the whole genome^4^ is colored in purple, known drug targets plus their direct interactors in yellow (P1), their interactors up to second-degree neighbors in orange (P2) and third degree neighbors in green (P3). The performance of the genes associated to the pathways in which the known drug targets are involved are colored in blue (F2). The vertical dashed line represents the average performance of the whole genome. The dashed curves represent the average Pearson correlation obtained with sets of random genes of the same size as the real selection of genes.

In a recent pharmacogenomics study a panel of 265 drugs was screened on 1001 cancer cell lines, for which gene expression, methylation, copy number alterations and single nucleotide variants were determined^4^. Most of the tested drugs (178) have known targets, which can be single proteins, groups of proteins, or, more broadly, pathways and cellular processes (e.g. DNA replication). However, we observed that the gene expression, quantified in 961 screened cell lines, of the known drug targets shows no correlation with the IC50 of the drugs on the same cells, meaning that the drug targets’ expression profiles possess poor capability of predicting the drug response (Fig. 1a). Despite gene expression showing the best predictive power in the original paper^4^, the effect of a drug on its target could also be altered by the pathogenic variants on the target protein (e.g. a mutation in the drug binding site). To some extent it is possible to predict the response to a particular drug by analyzing the state of germline and somatic variants^23^. However, we observed that for only 5 drugs out of 178 the presence of a pathogenic variant in the drug targets was found to be associated to resistance/sensitivity in the cell lines (Mann-Whitney U test, adjusted *p* < 0.05), number that increases to 10 when considering the 422679 mutations in the genome of all cancer cell lines (Mann-Whitney U test, adjusted *p* < 0.05, highlighted points in Fig. 1a). As observed in a previous study^18^, multi-gene predictors perform significantly better than single gene predictors, and the determination of the best combination of informative genes represents an ongoing and promising source of prediction improvement^19–21^. A first attempt used functional linked networks of genes centered on driver kinases in cancer cell lines, however the approach was focused on a small set of cell lines and drugs, reaching limited capability and performance^22^. We therefore tried to extend the analysis to the context of the drug targets, in terms of physical interactome^24^ and functional pathways^25^.

**Figure 2:**
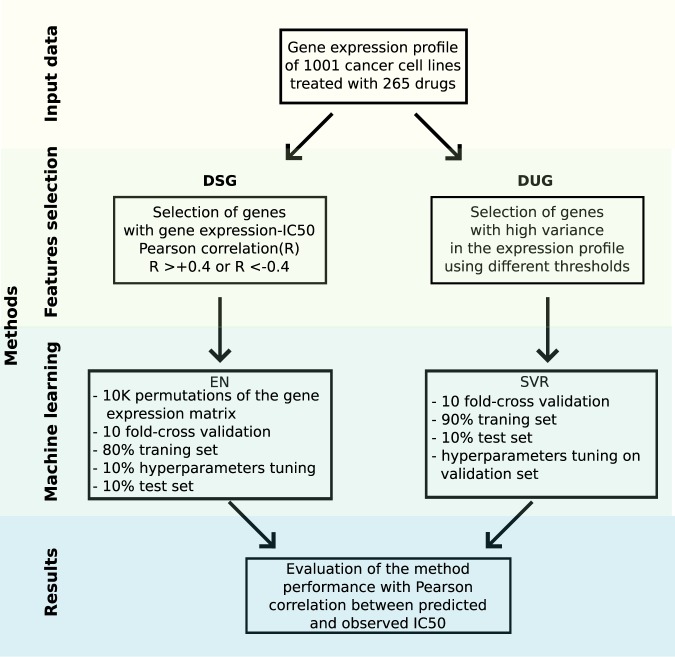
Experimental design of the DSG and DUG approaches. The Drug-Specific Genes (DSG) approach selects genes associated to the response of a particular drug, the gene expression of these genes are then used to train an Elastic Net Regression Model (DSG are selected in the training set only during the 10-fold cross-validation phase). The Drug-Unspecific Genes approach selects genes from initial dataset depending on the variance of their expression. Different number of genes to be used as features and different machine learning methods have been tested selecting the Support Vector Regression as the best method evaluated with a 10-fold cross-validation (hyperparameters are tuned in a validation set inside the training set). For both approaches the performance is evaluated with the Pearson correlation between the predicted and observed IC50 values (R_pred-obs_).

We applied an Elastic Net Regression (ENR), a linear modeling that solves the limitations of both LASSO and Ridge regressions that has been already successfully used for similar problems^5,9,10^, using the gene expression of a set of genes as features to predict the drug response in terms of IC50 (see Methods). The performance of ENR and of a non-linear approach, based on Random Forest, on this dataset has been already explored leading to comparable results^4^, with the former allowing a more straightforward interpretation of the models. The set of genes for which we computed the correlation between IC50 and gene expression profiles is firstly composed by the drug target and its first neighbors in its physical interactome (P1), then extended to the second (P2) and finally to the third neighbors (P3). As comparison we considered the whole genome, the set of genes for which the expression has been measured, as features of the predictive model, comparing the results with those obtained by Iorio *et al*.^4^, that reached an average Pearson R of 0.42 between experimentally measured and predicted IC50 (R_pred-obs_). Even though we observed that extending the neighborhood of the drug targets to second and third neighbors was improving the performance, these attempts reached a significantly lower R_pred-obs_ (0.22, 0.32 and 0.36 for respectively P1, P2 and P3 sets consisting of 23, 172 and 745 genes on average for each drug) than using the whole genome (Fig. 1b and Supplementary Table S1A). Moreover we observed a comparable performance with sets of random genes of the same size (Mann-Whitney U test, *p* ≥ 0.05, dashed lines in Fig. 1b), and this further demonstrates the paucity of information provided by the physical interactome around the drug targets in predicting its response. Another different, and possibly more biologically meaningful, selection of the analyzed genes associated to the drug targets could take into account the pathways in which they are involved. Hence, for each drug we tried separately (F1), and then merged into a unique gene set (F2), the genes in the associated pathways. We observed slightly better performances than selecting the genes from the physical interactome (R_pred-obs_ of 0.36 and 0.39 respectively for the F1 and F2 sets consisting of 87 and 737 genes on average for each drug, Supplementary Table S1A); the F2 set of genes reached better performance than the whole genome for 20 drugs out of the 178 considered in this analysis. However the whole genome remained the best predictive set of genes (Fig. 1b). A complete average performance comparison, using the same training and test sets in five 10-fold cross-validations after permuting the initial dataset, of all the combinations of gene sets (P1, P2, P3 and F2) and three different prediction methods (Elastic Net Regression, Random Forest and Support Vector Regression) can be found in Supplementary Table S1B. From this we can conclude that even though single genes can be found associated to drug response mechanisms, single-gene predictors based on either gene expression or presence-absence of variants in the drug targets cannot reliably predict the drug response in cancer cell lines. Moreover a multi-gene predictor based on the gene expression of known targets of anti-cancer drugs and on their physical/functional context, do not hold enough predictive power and is outperformed by a predictive model based on the whole genome. The fact that a random selection of genes allows to predict drug response with results comparable to the ones obtained with a selection of genes centered on the drug target is likely due to the fact that there are more genes regulating drug response other than the known drug targets and that an optimal gene combination able to predict a drug response must be sought with a more systematic approach. We designed two different approaches for the *a priori* selection of subsets of informative genes whose expression alone could provide a better estimate of IC50 values: i) an unspecific selection of genes to be used for every drug (termed DUG, Drug-Unspecific Genes), ii) a drug-specific subset of response-associated genes (termed DSG, Drug-Specific Genes) that can reflect drug peculiarities and different mechanisms of action and response (Fig. 2).

### Predicting drug response using Drug-Unspecific Genes

We considered the variance of the gene expression in order to reduce the number of genes used as features in a Drug-Unspecific Gene (DUG) predictive model (Fig. 2). We gave low priority to the genes with an expression that does not change significantly among the hundreds of different cancer cell lines and are therefore unable to discriminate well the different cancer cell line response. For each of the 17419 genes in the panel^4^ we ranked the variance of their expression profile in training cell lines. The top *n* genes (different *n* values were evaluated, as explained in the Methods section) were then used as features in a machine learning method to predict the IC50 for each tested drug in the test cell lines. The top 5000 genes were selected as the optimal gene set since adding 5000 additional genes did not result in a noticeable improvement in the R_pred-obs_ (0.43 ± 0.03 against 0.43 ± 0.03, Supplementary Table S2C) and in mean absolute error (MAE of 0.95 ± 0.03 against 0.95 ± 0.03) at the cost of doubling the number of genes, therefore diluting the genes more associated with drug response, and increasing the calculation time. The selected genes were then clustered by Pearson correlation (with a threshold of 0.8) of the gene expression IC50 profiles across the 1001 cell lines in order to remove redundancy. This resulted in a list of 4804 genes (DUG selected during the 10-fold cross-validation) that led to a performance of R_pred-obs_ 0.48 using a Support Vector Regression with Gaussian Kernel in a ten-fold cross-validation. Other tested methods, Random Forest and Elastic Net Regression, did not lead to optimal results (Supplementary Table S2C, Supplementary Table S2D for average performances using the same training and test sets in five 10-fold cross-validations after permuting the initial dataset). This final gene set is unspecific and has been used for all the drugs in the panel (drug-unspecific genes set, termed DUG, listed in Supplementary Table S2A). With this method 120 drugs out of 265 had a significantly better performance (t-test adjusted p < 0.05) than using the whole genome as feature set, which achieved a lower average R_pred-obs_ of 0.43.

For example, it was possible to have a great improvement in the prediction of the cellular response for drugs like Imatinib (R_pred-obs_ of 0.5 against the R_pred-obs_ of 0.34 reached using the whole genome, Supplementary Table S3). A functional enrichment analysis (see Methods) of this set of 4804 DUG revealed their involvement in general cellular functions, from the organization of the extracellular matrix, cell development and proliferation, to cell adhesion and migration and finally immune response (Supplementary Table S2B).

### Predicting drug response using Drug-Specific Genes

**Figure 3:**
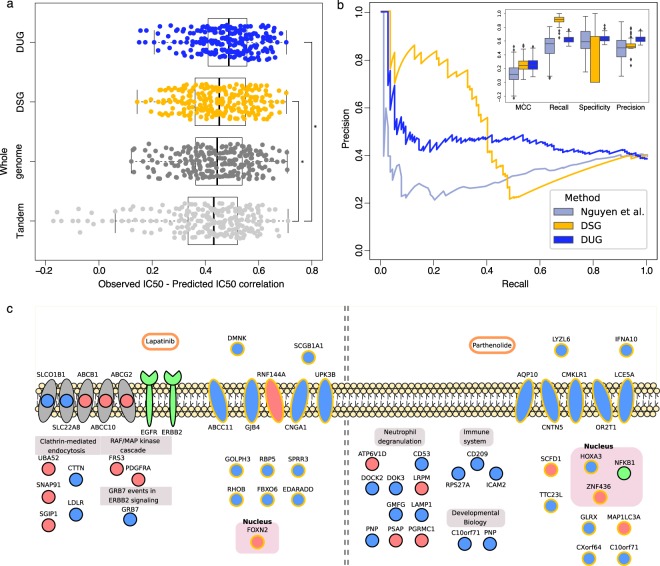
Comparison of different methods/approaches in predicting drug response. (**a**) Performance of different approaches in the prediction of drug response (IC50) measured as Pearson correlation between the experimentally measured and predicted IC50; TANDEM^9^ performance is colored in light grey, method described in Iorio *et al*.^4^ in dark grey, ENR-based DSG and SVR-based DUG approaches in yellow and blue respectively. Significantly different distributions of Pearson correlations are marked with a star (Mann-Whitney U test, *p* < 0.05). (**b**) Precision-Recall curve for the binarized drug response prediction for 120 drugs with the ENR-based DSG approach (colored in yellow), SVR-based DUG approach (colored in blue) and the method described in Nguyen *et al*.^18^ (colored in light grey), the performance of the three methods are reported in the inset boxplots as Precision, Recall, Specificity and Matthews Correlation Coefficient (MCC). (**c**) Schematic representation of Lapatinib and Parthenolide DSG localization in different cellular components; drugs are colored in orange and their known targets in green. Proteins belonging to the drug target pathways and selected as DSG are grouped by pathway and localization, “drug-unique” proteins are outlined with a golden color. Proteins are colored in blue or red if their gene expression has a respectively negative or positive correlation with the IC50 profile of the drug. The proteins in grey are known membrane transporters associated with the drug import or export (respectively blue and red)^26^.

**Figure 4:**
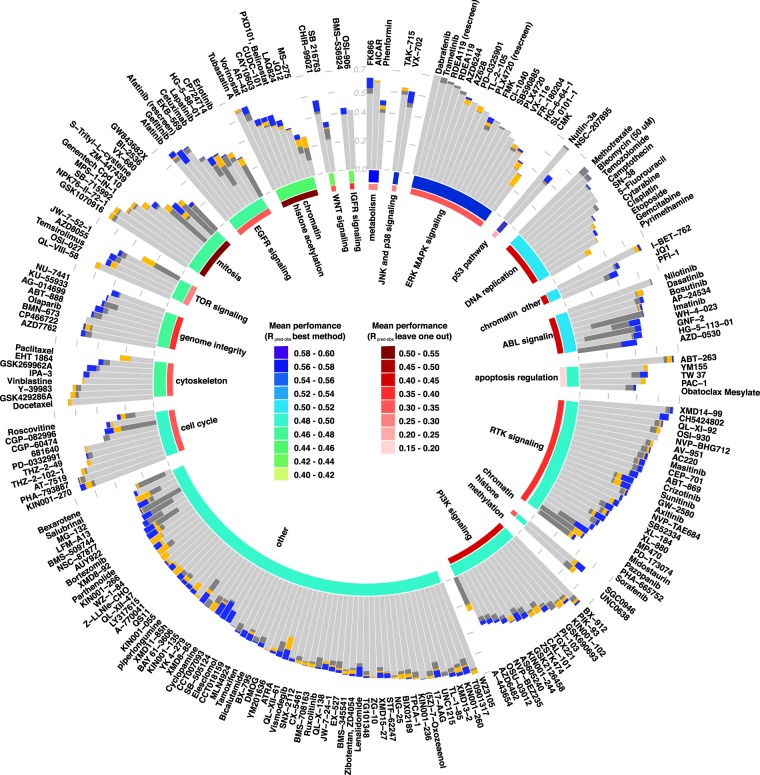
Breakdown of different method’s performance at single drug and drug class level. Performance (measured as Pearson correlation between experimentally measured and predicted IC50) of different methods for each analyzed drug in the dataset; TANDEM^9^ performance is colored in light grey, method described in Iorio *et al*.^4^ in dark grey, DSG and DUG approaches in yellow and blue respectively. Drugs are grouped in 21 functional groups (defined in the original study), and ordered by performance of the best method; for each drug the performances of the methods are ranked and displayed as overlapping barplot, in this way the improvement of the best methods is visible. The 21 groups are ranked clockwise by the average best prediction by the best methods, which is displayed by the inner circle (colored in a green to blue gradient). The red circle represents the results of the leave-one-out experiment (colored in a light to dark red gradient).

Although the previously described approach achieved a better prediction of the drug response with a reduced number of genes, their number is still too high to narrow down the list of pathways associated to the specific mechanisms of response to each drug, moreover the same subset of genes is used for every drug. We therefore created subsets of genes that are specific for each drug (termed Drug-Specific Genes, or DSG); these genes should be selected if they are observed as linked to the response of that particular drug over the whole set of cancer cell lines (Fig. 2). A DSG was then selected for a drug if its expression profile correlated (Pearson R > 0.4) with the IC50 profile of the drug over the portion of the dataset of cancer cell lines selected as training set (see Methods). The genes selected for a specific drug (DSG) were then used as features in the training of Elastic Net Regression (ENR) model^4^. The ENR was then tested on the cell lines in the test set (see Methods). We randomly permuted 10000 times the cell line dataset for each drug, therefore creating each time a slightly different set of selected genes, training and test sets, averaging out deviations due to cell line ordering and systematic errors. On average, we selected 558 genes for each of the 223 drugs for which it was possible to find drug-specific genes (Supplementary Table S4A) therefore reducing the number of features to be used in the machine learning and improving the prediction of the drug response over using the whole genome with an average R_pred-obs_ of 0.45 (Fig. 3a and Supplementary Table S3). While we observed an improvement in the prediction of drug response when using a SVR instead of an ENR with DUG, we observed no improvement with SVR coupled with DSG in terms of Rpred-obs (Mann-Whitney *p* < 0.05, Supplementary Table S4B for average performances using the same training and test sets in five 10-fold cross-validations after permuting the initial dataset). This performance is comparable to the DUG approach even though this one uses on average one order of magnitude less genes (Fig. 4), therefore prioritizing informative genes that, in our opinion, will be useful to guide hypothesis-driven experiments aimed at detailing all the cellular pathways involved in the response to a particular drug. In order to evaluate the robustness of the selected DSG we calculated the performance loss, in terms of R_pred-obs_, of each DSG set after removing every single gene, therefore assigning to each gene its contribution to the prediction of the response to a particular drug (Supplementary Table S5). We measured that on average, across the DSG sets, the performance loss was 0.02 R_pred-obs_ after removing the best gene in each DSG set.

### Improving the characterization of drug response mechanism

Lapatinib is a clinically approved drug which targets ERBB2 and EGFR in the treatment of breast cancer. We reached a R_pred-obs_ of 0.41 with the selection of 399 Lapatinib-specific genes, against a R_pred-obs_ of 0.16 obtained with TANDEM^9^ (a method described below), 0.31 obtained with the whole genome and 0.42 obtained with DUG (Fig. 4, respectively colored in yellow, light grey, dark grey and blue bars, and Supplementary Table S3). The selected informative genes are involved in the regulation of the immune system, inflammatory response, endothelial cell apoptotic process and mRNA metabolism and many encode for membrane transporters associated with drug import/export^26^. Only 7% of the genes associated to the cellular pathways involving ERBB2 and EGFR belong to the set of Lapatinib-specific genes, reducing the search space to fewer genes linked to the drug targets (Fig. 3c). For example GRB7 plays an important role in the signal transduction in response to EGF, promoting the activation of down-stream phosphorylation pathways (e.g. AKT1 and MAPK1) and its basal pre-treatment expression levels anticorrelate with the cellular response to Lapatinib not only in breast cancer cell lines (Pearson R = −0.64) but across the whole cell line panel (Pearson R = −0.39). In fact, its key role is demonstrated by its up-regulation after treatment with Lapatinib which, together with the rewiring of different signaling networks, and the inhibition of the HER2 signaling pathway, promotes cell survival and migration in breast cancer cell lines^27,28^.

Another example is Parthenolide, which is a drug in clinical development that targets NFKB1, and interferes with the assembly of the microtubule network^29^.

We reached a R_pred-obs_ of 0.38 with the selection of 565 Parthenolide-specific genes, against a R_pred-obs_ of 0.28 obtained with TANDEM, 0.28 obtained with the whole genome and 0.29 obtained with DUG (Fig. 4, respectively colored in yellow, light grey, dark grey and blue bars, and Supplementary Table S3). The functions of the 565 selected drug-specific genes are enriched in immune response, lymphocyte activation, leukocyte proliferation and somatic cell DNA recombination. Only 4% of the genes coding for proteins involved in the NFKB1 pathways were selected as DSG (Fig. 3c). ICAM-2, a key protein in leukocyte adhesion whose expression is targeted by NFKB1^30^, has been selected in over 99% of the DSG permutations tests for Parthenolide (showing a negative Pearson correlation of −0.33 across the whole cell line panel).

LRMP, a protein involved in the delivery of peptides to MHC class 1 molecules, and PSAP, among others, also show a strong association (selected over 99% of the permutation tests) to the response to Parthenolide with no experimental validation provided yet to the best of our knowledge. In both cases not only the new approach provided a significantly improved prediction of the drug effects but it also extended the list of genes to be prioritized for following experiments on genes involved in other cellular functionalities that are not directly linked to the known drug targets.

More generally, genes associated to the drug target pathway are not enriched in DSG genes coding for proteins involved in the pathways of the known drug targets more than random sets of genes of the same size (Mann-Whitney U test, *p* > 0.05). In fact only small percentages of the genes (around 5% on average, details for each drug in Supplementary Table S6A) involved in the pathways associated to the targets of respectively Lapatinib and Parthenolide were selected in their respective DSG sets. Additionally, we highlighted genes that showed a unique association (see Methods) to the response of one particular drug only, like Contactin-5 and Glutaredoxin-1 for Lapatinib and cGMP-gated cation channel alpha-1 and Dermokin for Parthenolide. We believe that these genes represent a valuable source of information as unique features of each drug response mechanisms that can set the ground for following hypothesis-driven experiments (complete list of “drug-unique” genes is available in Supplementary Table S6B).

### Different issues and approaches in the prediction of drug response

The task of predicting the drug response in cell lines comes with different issues, bound to the high dimensionality of the problem represented by the high number of genes and other features considered as features of the predictive model like mutations, methylation profiles and gene copy number alterations. Moreover the connection and information redundancy among these components and the continuous nature of the cellular response to external *stimuli* constitute an additional layer of complexity.

One approach for the reduction of the dimensionality of the problem has been proposed with the TANDEM method^9^, which predicts IC50 values for cell lines based on the integration of multiple data types, divided into upstream data (gene mutations, copy number alterations and methylation profiles and cancer type) and downstream data (gene expression), in order to improve the interpretation of the cellular response mechanisms. With TANDEM, an Elastic Net model is built using the upstream data, then the residuals are predicted with an Elastic Net built using gene expression. Upstream features are associated to drug response and their predictive binary value is calculated with a logistic regression using gene expression data. Therefore models give higher priority to upstream features, e.g. mutations and methylation status, that could explain the downstream gene expression resulting in more interpretable models of drug response.

We applied the TANDEM method to the 961 cell lines panel and measured an average R_pred-obs_ of 0.37 (Supplementary Table S3), which is significantly lower than both the DUG and DSG approaches (Mann-Whitney U test, *p* = 1.20 × 10^−7^ and *p* = 1 × 10^−3^ for DUG and DSG respectively) (Fig. 3a). Even though the authors included upstream data in the prediction no real improvement was reached compared to a predictive model based on gene expression only, stating that the information in upstream features is also present in gene expression profiles^9^. However the inclusion of upstream features is useful to recapitulate more complex gene expression patterns into, for example, few changes in gene mutations or methylation profile alteration could in principle improve the prediction and the characterization of the response mechanism. The comparison between single-gene-based and multi-gene-based predictors has been described in a recent work^18^, where the latter consistently outperformed the former. Moreover the authors explored the possibility to reduce complexity in the predictive model by defining binary cellular response (e.g. resistant/sensible) for 127 drugs. Since our approach predicts a IC50 value, we transformed this into a binary response (see Methods) in order to compare the two methods with the same metrics. Our approach reached higher Matthews Correlation Coefficient (MCC), with the SVR-based DUG and DSG approaches reaching MCC values of 0.27 and 0.25 respectively against 0.12 of the compared method. Both DUG and DSG approaches showed a better performance, for respectively 83 and 84 drugs out of 120, than the compared method. The DSG approach showed the best Sensitivity (0.9) compared to the DUG approach (0.6) and the other method (0.5), but showed lower Specificity values (0.31) compared to the DUG approach (0.65) and the other method (0.61) (Fig. 3b, Supplementary Table S3).

### Variability of the response mechanisms among similar drugs

One of the desired traits of a method able to predict the cellular response to a drug is the capability of predicting the effect of a novel, unscreened drug for which no experimental data is available yet. A first attempt could rely on the assumption that similar drugs have similar effects on the same cell. In this way, structurally similar drugs (or with similar active chemical groups) could share a similar IC50 profile across the cell line panel. We explored this possibility by correlating the similarity in the IC50 profiles of each pair of drugs (calculated as Pearson correlation) with the similarity of their chemical structure (see Methods and Supplementary Table S7) but we observed no significant association between the two measures. Drugs belonging to the same group, defined by the affected cellular pathway and described in the original work^4^, are not significantly more chemically similar than drugs belonging to different groups (Mann-Whitney U test, *p* > 0.05), on the other hand they share IC50 profiles across the cell line panel that are more similar than drugs of different groups (Mann-Whitney U test, *p* = 1.78 × 10^−40^). This highlights the issues in transferring drug response from one drug to another using chemical similarity, as it has been observed previously in other studies^31–33^. When the drugs were grouped depending on their chemical similarity, e.g. with a similarity score (see Methods) equal or higher than 0.7 and equal or lower than 0.3, the similar drugs were showing higher IC50 profile correlation than non-similar drugs (Mann-Whitney p < 0.05). Several studies^34–37^ showed the relationship between chemical similarity and cellular response similarity, therefore the reasons behind the low overall correlation observed in our dataset are most likely due to the relatively small size and diverse nature of the original drug datasets, especially when compared to much bigger and diverse datasets used in proteochemometrics studies.

We therefore explored the extent to which a drug class-derived ensemble model could be used to infer the response mechanism of a single drug of the same class. The 265 drugs in the initial work by Iorio *et al*.^4^ were grouped by targeted process/pathway into 21 classes, each of which has been analyzed to test the ability of a class-derived prediction model in predicting the cellular response to an “unknown drug” that could belong to the same class. To simulate this setting, we used a leave-one-out test in which the response to the drug being left out was iteratively predicted using the DSG sets of all the other drugs in the class. The predictions of all the drugs in a class are then averaged out and the prediction performance on the tested drug left out is then evaluated with the R_pred-obs_. We measured a mean R_pred-obs_ value of 0.35 among the different classes, concluding that it is possible to transfer, to a certain degree, the response mechanisms among drugs that are supposed to aim at the same or similar targets, using different metrics and parameters as proxy. However two drug classes, targeting proteins involved in “mitosis” and “chromatin histone acetylation” associated pathways, reached high performances with predictions that were comparable to those obtained using the DSG sets of each specific drug (R_pred-obs_of 0.55 and 0.53 respectively).

## Discussion

Different approaches have tried to leverage genomic data in order to predict the effect of therapeutic drugs on cancer cell lines. Many methods have been developed to combine different sources of information or to reduce the search space by removing redundant information in the predictive models.

The whole genome has been used previously to predict the cellular response to chemical compounds, although limited in the prediction performance and in the selection of informative genes that could explain resistance/sensitivity mechanisms. Consequently, we explored the possibility of reducing the number of informative genes employed in the prediction of pan-cancer drug response, both in a drug-unspecific (i.e. the same for all the drugs in the panel) and drug-specific fashion employing different pre-processing and machine learning approaches and gene expression data. We observed a significant improvement in the prediction of the drug response in a panel of 1001 cell lines screened with more than 200 drugs, reaching better performance than other already published methods with both a drug-unspecific and a drug-specific approach. We also showed how this was possible with a reduced number of genes (on average two orders of magnitude lower) compared to a previous method using the whole genome^4^. This has the double advantage of reducing the noise in the predictive model and prioritizing the genes and of the cellular mechanisms behind the response to a particular drug, which was not limited to the known drug targets.

Interestingly, we demonstrated how the gene expression of the known drug targets and their variants, introduced in different cancer types, do not hold enough predictive power and cannot be used alone in the prediction of the effect of their drug. Therefore the genes coding for drug-specific response have to be selected from a wide array of pathways, in fact only a small fraction of the genes coding for proteins in the pathways involving the known drug targets are selected as drug-specific genes.

Finally, we explored the variability in the response mechanisms of different classes of drugs as defined by our model. Even though drugs in the same class do not usually share the same chemical structure they do share similar drug response profiles across different cell lines. We observed that despite this variability, generalized drug class-derived and reliable predictive models can be generated, a features that would greatly contribute to the analysis of novel unknown drugs.

While our performances demonstrate the usefulness of both drug-unspecific and drug-specific approaches, using significantly less data nonetheless, there is still room for further improvements. Additional information from other sources of data (e.g. methylation, mutation profiles, non-coding RNA quantitative data) and integration of other dynamic measures, e.g. the activity and relationship of transcription factors^38^, could provide tissue-specific traits in the response mechanisms. It must be noted also that cell lines do not perfectly reflect primary tumor samples and the translation of these predictive models will require the integration of clinical data, which is sparse for a limited number of drugs and non-standardized at the current stage. The inclusion of *in vivo* patient-derived gene expression and drug response measurements in machine learning approaches is a step towards real cases moving from *in vitro* cancer cell lines-derived measurements^19^. The reduction in the number of features (genes, mutations and components of other types of data) is a current area of drug response prediction improvement^19–21^.

Finally, since proteins are the first effectors of a cellular response, using only transcriptomics data to reliably predict drug response in a particular cellular condition can only provide an incomplete picture, since translation regulation can be complex and post-translational modifications can modulate the protein cellular roles. Recently different studies tried to use proteomics data to model drug response and identify possible biomarker pathways, although on very limited datasets^39,40^. Even though proteomics data is less abundant^41^ compared to genomics and transcriptomics data, we think that the integration of proteomics data, for example protein abundance and proteoform status (e.g. epigenetic markers, post-translational modifications and their stoichiometry and occupancy status), will constitute the next big step forward in the field.

## Methods

### Dataset

Gene expression data and cell lines drug response data used in this study were generated in a previous work by Iorio *et al*.^4^. The dataset consists of large-scale genomic data, including gene expression and genomic variant profiles, copy number variations and methylation for 1001 cancer cell lines representing 29 different tissues. The basal expression profile for each cell line was obtained using a microarray analysis (A-GEOD-13667 - [HG-U219] Affymetrix Human Genome U219 Array). The raw expression data of each gene has been normalized with z-scores in each cell line. On average each cancer type is represented by 30 cell lines and each cell line has been treated with different drugs (265 drugs in total). Drug response is expressed in terms of IC50, which is the drug concentration that reduces viability by 50% *in vitro*. Lower values of IC50 are associated with a higher sensitivity of a cell line to a given drug and vice versa.

### Selection of genes associated to known drug targets

In the first part of the study we used the known drug target to build gene sets representing the physical and functional environment of the targeted molecular processes. The drug target was known for most of the cases (178 of the drugs had one or more known targets, while the remaining 87 drugs were associated to the description of the cellular processes hit by the drug)^4^. We used the STRING database^24^ to build networks centered around the known drug target and including up to first (direct interactions, P1 gene sets), second and third degree interactors (indirect interactions, respectively P2 and P3 gene sets). We selected only experimentally obtained physical protein-protein interactions with STRING scores higher than 0.9. The target proteins were annotated only for 178 drugs out of 265, for which the described gene sets were created. The remaining drugs, for which only the general affected cellular processes (e.g. DNA replication) were described, were not considered in this particular analysis. In order to build the functional (and partly physical) neighbors of the drug targets, we used the REACTOME database^25^, selecting the genes involved in the pathways of the 178 drug targets (F1 gene sets for single pathways and F2 gene sets for merged pathways associated to the same drug target). These gene sets are reported in the Supplementary Table S1A. We then used two different approaches to select genes that could better describe the drug response regardless of the annotated known targets.

### Selection of drug-unspecific genes

Genes were ranked according to their variance in gene expression across the cancer cell line dataset were selected and the top genes were selected. An equal number of random genes were selected as control. We selected an optimal number of feature genes by exploring different sizes of the gene sets for a Random Forest learning method: 25, 100, 1000, 5000 and 10000. We tested the learning method in a 10 random split cross validation where 90% of the data was used for training and the remaining 10% was used as test for the prediction of the IC50 values; both the number of selected genes and the variance threshold were treated as hyper-parameters and the best combination was chosen in a validation set inside the training set with a 5-fold cross-validation. Results were evaluated in terms of Pearson correlation between predicted and observed IC50 (R_pred-obs_). The selected genes were then clustered by Pearson correlation (with a threshold of 0.8) of their gene expression profiles across the cell lines (complete gene list is reported in Supplementary Table S2A).

### Drug-unspecific gene-based machine learning

The 4804 selected drug-unspecific genes are then used as features in a 10 random split cross validation, using Support Vector Regression (SVR)^42^, where 90% of the data was used for training and the remaining 10% was used as test; again, the best combination of model hyperparameters were chosen in a validation set inside the training set. Performance was estimated through Pearson correlation between the predicted and observed IC50 values and drugs with significantly better performances were identified with a *t*-test with 0.05 as significance threshold.

### Selection of drug-specific genes

In an alternative approach, for each drug, we selected the genes whose pre-treatment expression profile correlated, or anticorrelated, with the drug IC50 profile (Pearson correlation equal or higher than +0.4 or equal or lower than −0.4). In order to select a gene as informative for a particular drug we sorted the expression profile matrix and selected only 10% of cell lines with the highest expression of the considered gene and 10% with the lowest expression of the same gene. We then correlated the expression profile of the analyzed gene in this selected 20% of the cell lines with the IC50 profile of the considered drug in the same cell lines. In this case, each screened drug was associated to a specific set of informative genes (Supplementary Table S4A) that are involved in the response mechanisms (drug-specific gene set, termed DSG).

### Drug-specific gene-based machine learning

We used the Elastic Net Regression (ENR), using the *glmnet* R package^43^ for the drug-specific gene sets, a model also described in a previous work by Iorio *et al*.^4^. Gene expression values were scaled to have zero mean and unit standard deviation. In order to make a fair comparison between the ENR in this study and the original work by Iorio *et al*. we selected 80% of the dataset to train the model and 10% for the test and prediction of the IC50 values of a given drug, as the remaining 10% was originally used for the parameters optimization. We performed 10000 permutations of the cell lines in the panel before the training and test phases in order to avoid biases in the initial dataset. The selection of the DSG genes has been performed each time on the cell lines belonging to the training set only, removing redundancy between training and test set. Finally, we compared the predicted and experimentally observed IC50 using the Pearson correlation.

### Functional enrichment

The functional enrichment analysis was made using GOrilla^44^ and Revigo^45^. We focused on Biological Process and Cellular Component GO term categories, providing the whole genome as background. We then used REViGO to summarize and visualize the enriched GO terms identified with GOrilla.

### Drug similarity

The chemical structures of the drugs were collected using the identifiers provided in the original paper by Iorio *et al*. Drug names were converted in Compound identification number (CID) using the PubChem database^46^. SMILES chemical representations and sdf files were downloaded using PubChem programmatic access option. We used different approaches for the estimation of similarity between drugs. Structural similarity between drugs was calculated through the Tanimoto and Tversky coefficients based on atom pair descriptors, atom pair fingerprints and Mismatch Tolerant Maximum Common Substructure Detection using ChemmineR^47^ and drug effectiveness similarity has been estimated through Pearson correlation, with complete observations, of the IC50 profiles of the two compared drugs on 1001 cell lines.

### Binarization of the predicted drug response

Our predicted IC50 response values were converted into binarized values (e.g. 0 and 1 values for cell lines respectively resistant and sensible to a particular drug) in order to provide a fair comparison with a binary drug-response classifier developed by Nguyen *et al*.^18^. The classification was done by using the median value of the drug’s IC50 distribution on 1001 cell lines as reference. IC50 values higher than the median were labeled as resistant and lower values labeled cell lines as sensible.

### Drug-unique genes identification

We have selected a set of “unique” genes for each drug from its pool of drug-specific genes. Given a gene, the number of cell line permutations in which the gene is selected as informative for each drug in the panel is calculated (Supplementary Table S4A) and normalized as z-score. The z-scores of all the drugs in the panel are then sorted and the gene is deemed “unique” for the first ranked drug if its z-score is equal or higher than 3 and if the second-ranked drug has a z-score that is lower than 3.

## Supplementary information

Supplementary Materials

Supplementary Table S1

Supplementary Table S2

Supplementary Table S3

Supplementary Table S4

Supplementary Table S5

Supplementary Table S6

Supplementary Table S7

## Data Availability

The scripts generated in this work are available at https://github.com/lucaparca/dre. Code is written in R, initial data is provided and already pre-processed (gene expression normalized with z-scores in each cell line) and ready for the analysis. An *n* number of permutations of the cell line gene expression dataset can be specified for different dataset partitioning into training and test sets. Prediction for specific drugs are then provided as results with the mean and standard deviation of the performance (Pearson correlation between observed and predicted IC50).
