# Supplementary material for: Modeling cancer drug response through drug-specific informative genes
Source: Sci Rep. 2019 Oct 23;9:15222. doi: 10.1038/s41598-019-50720-0 (PMC6811538; doi:10.1038/s41598-019-50720-0)
Supplement: Supplementary file 1 — Supplementary Materials [file 41598_2019_50720_MOESM1_ESM.pdf]

TITLE

Modeling cancer drug response through drug-specific informative genes

AUTHORS

Parca Luca[1†], Pepe Gerardo[1†], Pietrosanto Marco[1], Galvan Giulio[2], Galli Leonardo[2], Antonio Palmeri[1§], Sciandrone Marco[2], Ferrè Fabrizio[3], Ausiello Gabriele[1], Helmer Citterich Manuela[1*]

[1]Department of Biology, University of Rome "Tor Vergata", Rome, Italy.

[2]Department of Information Engineering, University of Florence, Florence, Italy.

[3]Department of Pharmacy and Biotechnology, University of Bologna Alma Mater, Bologna, Italy.

[§]Present address: Celgene Institute for Translational Research Europe, Sevilla, Spain

[*]Correspondence to manuela.helmer.citterich@uniroma2.it

[†]The authors wish it to be known that, in their opinion, the first 2 authors should be regarded as joint First Authors.

SUPPLEMENTARY INFORMATION

Supplementary Table S1. **A**) List of the drugs analyzed in the paragraph "Contribution of known drug targets and of their known interaction partners to the cellular drug response". Drugs are reported together with their known targets and the proteins associated to their known targets using protein-protein interaction and pathway data. **B**) Comparison of average performances of different combination of gene sets (P1, P2, P3 and F2) with 3 different prediction methods (Elastic Net Regression, Random Forest and Support Vector Regression).

Supplementary Table S2. **A**) List of genes selected as Drug-Unspecific Genes (DUG). **B**) Gene Ontology terms enriched in DUG. **C**) Performance of machine learning methods with different variance-based selection of genes. **D**) Average performance of DUG with different machine learning methods (Elastic Net Regression, Random Forest and Support Vector Regression) using the same training and test sets in five 10-fold cross-validations after permuting the initial dataset.

Supplementary Table S3. Performance comparison in the prediction of drug response in cancer cell lines between the proposed approaches and other published methods.

Supplementary Table S4. **A**) Selection of Drug-Specific Genes (DSG) across the cell line panel. Each row represents a single gene (gene name is reported) and each column represents a drug in the screening panel. For each gene the number of times it gets selected for a specific drug during the 10000 permutation test is reported. **B**) Average performance of DSG with different machine learning methods (Elastic Net Regression, Random Forest and Support Vector Regression) using the same training and test sets in five 10-fold cross-validations after permuting the initial dataset.

Supplementary Table S5. Contribution of each gene in the DSG set for a specific drug measured as the loss in terms of Pearson correlation between predicted and observed IC50

values ($R_{pred\text{-}obs}$). Only genes (rows) selected at least once in a DSG set are shown; genes with a value of 0 are not part of the DSG set for that particular drug (columns).

Supplementary Table S6. **A**) Intersection between the DSG of each drug and the genes coding for protein in the pathways associated to the known targets of the drug (gene set F2 in Supplementary Table S1). **B**) Drug-unique genes selected for each drug in the dataset.

Supplementary Table S7. PubChem IDs and SMILE representation of the structure of the drugs analyzed in the paragraph " Variability of the response mechanisms among similar drugs".
